# Micronutrient adequacy is poor, but not associated with stunting between 12-24 months of age: A cohort study findings from a slum area of Bangladesh

**DOI:** 10.1371/journal.pone.0195072

**Published:** 2018-03-29

**Authors:** Kazi Istiaque Sanin, M. Munirul Islam, Mustafa Mahfuz, A. M. Shamsir Ahmed, Dinesh Mondal, Rashidul Haque, Tahmeed Ahmed

**Affiliations:** 1 Nutrition and Clinical Services Division, International Centre for Diarrhoeal Disease Research, Bangladesh (icddr,b), Dhaka, Bangladesh; 2 Renal Unit, Menzies School of Health Research, Darwin, Australia; 3 Infectious Diseases Division, International Centre for Diarrhoeal Disease Research, Bangladesh (icddr,b), Dhaka, Bangladesh; TNO, NETHERLANDS

## Abstract

The prevalence of stunting among children below 5 years of age is higher in the slum-dwelling population of Bangladesh compared to that in both urban and rural areas. Studies have reported that several factors such as inadequate nutrition, low socio-economic status, poor hygiene and sanitation and lack of maternal education are the substantial predictors of childhood stunting. Almost all these factors are universally present in the slum-dwelling population of Bangladesh. However, few studies have prospectively examined such determinants of stunting among slum populations. In this paper, we reveal the findings of a cohort study with an aim to explore the status of micronutrient adequacy among such vulnerable children and establish its association with stunting along with other determinants. Two-hundred-sixty-five children were enrolled and followed since birth until 24 months of age. We collected anthropometric, morbidity and dietary intake data monthly. We used the 24-hour multiple-pass recall approach to collect dietary intake data from the age of 9 months onward. Micronutrient adequacy of the diet was determined by the mean adequacy ratio (MAR) which was constructed from the average intake of 9 vitamins and 4 minerals considered for the analysis. We used generalized estimating equation (GEE) regression models to establish the determinants of stunting between 12–24 months of age in our study population. The prevalence of low-birth-weight (LBW) was about 28.7% and approximately half of the children were stunted by the age of 24 months. The average micronutrient intake was considerably lower than the recommended dietary allowance and the MAR was only 0.48 at 24 months of age compared to the optimum value of 1. However, the MAR was not associated with stunting between 12–24 months of age. Rather, LBW was the significant determinant (AOR = 3.03, 95% CI: 1.69–5.44) after adjusting for other factors such as age (AOR = 2.12, 95% CI: 1.45–3.11 at 24 months and AOR = 1.97, 95% CI: 1.49–2.59 at 18 months, ref: 12 months) and sex (AOR = 1.98, 95% CI: 1.17–3.33, ref: female). Improving the nutritional quality of complementary food in terms of adequacy of micronutrients is imperative for optimum growth but may not be adequate to mitigate under-nutrition in this setting. Further research should focus on identifying multiple strategies that can work synergistically to diminish the burden of stunting in resource-poor settings.

## Introduction

Globally, chronic undernutrition manifested by stunting (height or length-for-age Z-score < -2SD) affected 159 million children under five-year-old in 2014 [1]. Stunting is a huge burden particularly for the low and middle-income countries (LMICs) [2]. Asia remains the continent with the highest number of stunted children (approximately 100 million) while, in Africa, the prevalence remains stagnant at around 40% [3]. While a defined pathway of stunting remains ambivalent, it is presumed to be an outcome of complex interactions among diverse factors, such as inadequate nutrition, repeated infection, intrauterine growth restriction [4,5] and so on. A significant proportion of stunting in LMICs occurs during 6–24 months of age [6,7], a period when complementary foods are introduced in children’s diet. During this time, the nutritional requirement becomes much higher due to both physiological and pathological factors such as rapid growth, limited gastric capacity and frequent exposure to pathogens [8]. To meet this increased demand, optimum breastfeeding, as well as a wider range of safe and nutrient-dense foods must be provided [9] for appropriate physical growth and neurodevelopment [8].

Unfortunately, the diets of infants in LMICs are predominantly cereal based and a majority of them consume diluted family foods with lower nutrient densities [9]. Such complementary foods usually lack inclusion of animal protein sources and subsequent adequacy and bioavailability of several micronutrients such as vitamin A, zinc, iron, which are critical for growth [10]. As a result, it has been estimated that more than half of the preschool children are anemic and approximately 140 million preschool children have subclinical vitamin A deficiency [11]. Furthermore, it has been postulated that about half the world’s population is at risk of zinc deficiency [12].

Over the last decade, Bangladesh has successfully achieved the majority of the millennium development goals [13,14]. Yet, undernutrition is still prevalent in the country [15]. Recent national demographic and health survey reports that prevalence of stunting, wasting and underweight in under-5 children are 36%, 14%, and 33%, respectively [16]. According to UNICEF, Bangladesh is one amongst twenty countries in the world with the highest burden of stunting in under-5 children [17]. Several factors such as inadequate infant and young child feeding (IYCF) practices [18], poor dietary diversity and low micronutrient density of complementary foods [19,20], repeated infection [21] have been reported contributing to such high burden of childhood undernutrition in the country. However, most of these findings are based on cross-sectional surveys focusing on rural population rather than those residing in slum areas.

Previously, undernutrition was assumed to be a concern for rural impoverished areas solely. In recent times, rapid urbanization has led to a change in the broader scenario. Now undernutrition is considered a malady, affecting a substantial section of poor and underprivileged community living in the slum areas [22]. Approximately 2.2 million people are currently living in 14,000 Bangladeshi slums [23]. Children living in such areas are the most disadvantaged as the prevalence of stunting is greater among these children compared to the overall urban average (40% vs 26%) [17]. It has been hypothesized that poor dietary intake resulting in micronutrient inadequacy could be attributable to suboptimal linear growth, hence stunting. However, there is a paucity of studies where the association between stunting and dietary intake during complementary feeding period has been explored using a longitudinal approach to establish a causal relationship. Therefore, this paper reports the findings of a study where we followed a cohort of children, measured their dietary micronutrient intake and adequacy, and conducted statistical analyses to unveil any causal relationship between micronutrient adequacy and stunting along with other determinants in a slum setting in Dhaka, Bangladesh.

## Methodology

### Study site and recruitment

#### Study design

This study is part of an international, multidisciplinary, multi-country study known as the MAL-ED study that focuses on the interaction between enteric infection and malnutrition [24]. Bangladesh is one of the field sites among the eight countries [25]. This MAL-ED study consists of three integral components: (1) birth cohort component, (2) case-control component, and (3) twin children studies. Data for this paper have been generated from the Bangladeshi birth cohort component.

#### Study site

The enrolled participants reside in an underprivileged community in Mirpur, Dhaka, Bangladesh, which is one of the 21 administrative units of the nation’s capital. This site was selected considering the poor socioeconomic status of its inhabitants, and subsequent compromised sanitary condition typical of any congested urban settlement. The details of the study site have been reported elsewhere [25].

#### Study participants

Total two hundred and sixty-five healthy newborn children were enrolled in the birth cohort component between February 2010 and February 2012. To facilitate enrollment in the cohort study, a census was carried out to identify pregnant women within the community during the study period. Eligible participants were identified applying specific inclusion and exclusion criteria. The inclusion criteria included healthy infants enrolled within 17 days of birth, caregiver had no plans to move out of the catchment area for at least 6 months following enrollment and the willingness of the caregiver to be visited in their home monthly. Exclusion criteria were the family had a plan to move out of the catchment area for more than 30 consecutive days during the first 6 months of follow-up, maternal age <16 years, not a singleton pregnancy (i.e., twins or triplets), presence of another child being already enrolled in the MAL-ED study, severe illness requiring hospitalization prior to recruitment and severe acute or chronic conditions diagnosed by a physician (e.g., neonatal disease, renal disease, chronic heart failure, liver disease, cystic fibrosis, congenital conditions etc.).

### Data collection procedure

Upon enrollment, each child's date of birth, sex, and birth weight (if available) were recorded. Information about the initiation of breastfeeding was noted and the child's length, weight, and head circumference were measured. Information regarding dietary intakes of these children was collected monthly beginning at 9 months until 24 months of age. Anthropometric and morbidity data were also collected routinely on a monthly basis.

#### Procedure of dietary assessment

The quantitative dietary intake data of the children were collected monthly on non-consecutive days using the 24-hour (24-hr) multiple-pass dietary recall approach. Such an approach represents an ideal technique to quantify the energy and nutrient intakes of young children over time [26]. As we have 16 monthly dietary data available for each child from 9 to 24 months, these were combined to indicate a more reliable information regarding typical intake [27] over the interval of 9–24 months or over the specified time points (e.g., 9–12, 13–18, or 19–24 months) as described in the original design of the study [28]. The results of average intakes between 9–12, 15–18, 21–24 months and overall 12–24 months period have been calculated, analyzed and presented here. Averaging the dietary data from four 24-hr recalls has allowed to estimate the usual intake of micronutrients as multiple 24-hr recalls (3 or more recalls) are more accommodating for establishment of an association between diet and disease [29,30].

Clinical research staff (trained by experienced dieticians) conducted the 24-hr recall with multiple-pass [31] interviewing method using visual aids. They used standardized household measuring utensils and food pictures of various portion sizes to assist the mothers in quantifying the dietary intake of their children in preceding 24 hours. The 24-hr recall was conducted on non-consecutive days with no prior notification. The interviews were conducted in such a way so that, out of every 4 recalls, 1 would be scheduled on Friday which is the weekend in Bangladesh. The dietary intake data were converted to nutrients using a locally adapted food composition table (Table 1). To ensure the quality of the data collection, 10–20 secondary dietary recalls with randomly selected participants were performed monthly.

**Table 1:** Sources of food composition table.

1. USDA20-US Department of Agriculture Standard Reference Version 20 (2007)
2. World Food Dietary Assessment System, UC Berkeley
3. Food and Nutrient Database for Dietary Studies, Version 3.0 (2008)
4. NDS-Nutrient Data System for Research
5. Manufacturer label
6. Recipes based on information collected from homes or from local recipe books

### Measurement of nutritional adequacy

To measure the overall nutritional quality of the diet, we used mean adequacy ratio (MAR) as an index [32]. Among multiple indices, the MAR has been found to exhibit a positive association with various health indicators in previous studies [33,34]. We calculated the MAR from the nutrient adequacy ratios (NARs) of thirteen micronutrients (9 vitamins and 4 minerals) with the vitamins being: vitamin A, vitamin E, thiamine, riboflavin, niacin, folate, vitamin B6, vitamin B12, vitamin C, and minerals being: calcium, iron, zinc, and copper. The NAR for any given nutrient is the ratio of a person’s average daily intake and the recommended dietary allowance (RDA) of that specific nutrient, accounting for the individual’s sex and age [35,36]. The age-specific RDAs have been adapted from the recommendation developed by the Institute of Medicine (IOM) [37]. After calculating NARs of each of the individual micronutrients, the ratios (Intake/RDA) which were greater than 1 were truncated to 1 so that nutrients with high NAR cannot compensate for nutrients with low NAR [38]. The MAR was calculated as the sum of the NARs (truncated to 1) divided by the total number of nutrients (13 micronutrients in our case)

$$MAR_{x}=\sum (Intake_{x}/RDA_{x})/13$$

Here intake_x_ is the daily average intake of a nutrient X, and RDA_x_ is the recommended dietary allowance for that nutrient. As in previous studies [39], the calculated MAR was not weighted to signify the degree of importance of different nutrients or to correct for the differences in bioavailability. Therefore, an MAR of one represents equal or greater intake than the recommended value for all nutrients, and conversely, an MAR below one indicates lower than the recommended intake for one or more nutrients.

### Anthropometric data

Trained field workers obtained the children’s weight with minimum clothing using a frequently standardized digital scale with a 10-g precision (Seca, model-345, Hamburg, Germany) and recumbent length using an infantometer (Seca, model-416, Hamburg, Germany) with a precision of 1 mm. The new WHO growth standard (2006) [40] was used to calculate different anthropometric indices: weight-for-age Z-score (WAZ), length-for-age Z-score (LAZ) and weight-for-length Z-score (WLZ).

### Operational definitions

**Low birth weight:** Weight at birth of less than 2,500 grams.

**Stunting:** length-for-age Z score (LAZ) less than minus two standard deviations of the WHO median Child Growth Standard.

### Variable selection for analysis

We selected the variables based on determinants of stunting reported in the previous literature from LMICs [41,42] and availability of data from our study. Different tiers of determinants of stunting, such as inherent, proximal, intermediate and distal, have been described in various published studies. The variables of interest for our study are provided according to those tiers in Table 2.

**Table 2:** Variables of interest.

Factors	Explanatory/independent variables (between 12–24 mo age)	Outcome/ dependent variable (between 12–24 mo age)
Inherent ^a^	Age, sex	Stunting ^b^
Proximal ^b^	Nutritional quality (MAR%)^c^, current breastfeeding status, morbidity status (diarrhea)	
Intermediate ^a^	Maternal age, birth weight, birth order, presence of improved toilet, drinking water source	
Distal ^a^	Maternal education, household asset index	

^a^ Information was collected during enrollment

^b^ Information was collected monthly between 9–24 months of age. Due to longitudinal nature, data have been summarized at 12, 15 and 24 months of age and used in the generalized estimating equation regression that provided relationship between outcome and explanatory variables for overall 12–24 mo of age.

^c^ MAR- Mean adequacy ratio of vitamins and minerals

Due to longitudinal nature of the data, we categorized the age variable into 3 stages as 9–12, 15–18 and 21–24 months. Nutrient intake data were averaged over these time periods and the mean intake was analyzed and reported from four 24-hour recalls. Nutritional adequacy was presented as MAR and expressed as a percentage. Current breastfeeding status was categorized as breastfed or non-breastfed depending upon the information collected during the last 24-hr recall at 12, 18 and 24 months of age. Similarly, the morbidity status was categorized into a binary variable depending upon a child having a diarrheal episode in the 15 days prior to the monthly interview at 3 respective time points. Birth weight was categorized as the history of LBW or not. A toilet with flush capability was coded as an improved toilet and the household asset index was constructed using household asset data obtained from the socio-economic status (SES) questionnaires. Principal components analysis was performed to produce a common factor score for each household, and depending on the score, households were categorized into low (poor), intermediate, and high (wealthy) households. Finally, stunting was selected as a binary outcome variable at 12, 18 and 24 months of age.

### Ethical consideration

The study was approved by the Research Review Committee and Ethical Review Committee of the International Centre for Diarrheal Disease Research, Bangladesh (icddr,b), Dhaka, Bangladesh. All the investigators obtained the certificate for “Human Participants Protection Education for Research Teams” through an online course sponsored by the National Institutes of Health. Written consent was given by the mothers or legal guardians of the children. During each follow-up visit, mothers or primary caregivers provided verbal consent on behalf of the children. All information, research data, and related records were anonymously entered into a computer and were analyzed with no indication of the child’s name or identity.

### Data analysis

SPSS for Windows (IBM SPSS Statistics V22.0) and WHO Anthro software (Geneva, Switzerland) were used to analyze the data upon entry. The mean and standard deviation are reported for continuous variables and frequency distribution for categorical variables. Continuous variables with normal distribution were compared between groups using Student’s t-test after verifying the equality of variance (Levene’s test). The difference in proportion was compared using a Chi-square test or the Fisher’s exact test if the expected number in any cell was < 5.

We investigated multicollinearity between independent variables using a correlation matrix and variance inflation factor (VIF) values. We used the collinearity diagnostics from linear regression command as collinearity statistic in regression represents the relationship among the predictors ignoring the type of dependent variable [43]. Predictor variables with a VIF value of greater than five were screened again for correlation and only the variables of interest were kept in the model avoiding collinearity.

As we have collected repeatedly measured data from the same child over months, to investigate the association between outcomes and predictor variables, generalized estimating equation (GEE) regression was used. The outcome of interest was the development of stunting between 12–24 months of age as a binary categorical variable. GEE method focuses on average changes in response over time and the impact of covariates on these changes. Therefore, the reported odds ratio is a pooled odds ratio of effect (stunting) of all the predictors over 12–24 months of age. We presumed an autoregressive (1) (AR(1)) covariance matrix with robust variance estimates. Categorical predictor variables (age, gender, LBW or not, breastfeeding status, morbidity status, mother’s education level, the presence of improved toilet, water source, and household asset index) were selected as factors and continuous variables (mean adequacy ratio, maternal age, and birth order) were treated as covariates. Cases with missing values in terms of the dependent variable, covariates, and factors were excluded during analysis. Initially, bivariate analysis was performed to identify the unadjusted effect of each predictor on stunting through individual GEE model. In subsequent models, all the predictor variables were entered simultaneously to obtain the adjusted final model. The model goodness of fit was determined based on the lowest quasi-likelihood under independence model criterion (QIC) value. A probability of less than 0.05 was considered statistically significant and the strength of association was determined by estimating the adjusted odds ratios (AOR) and their 95% confidence intervals (CIs).

## Results

**Fig 1:**
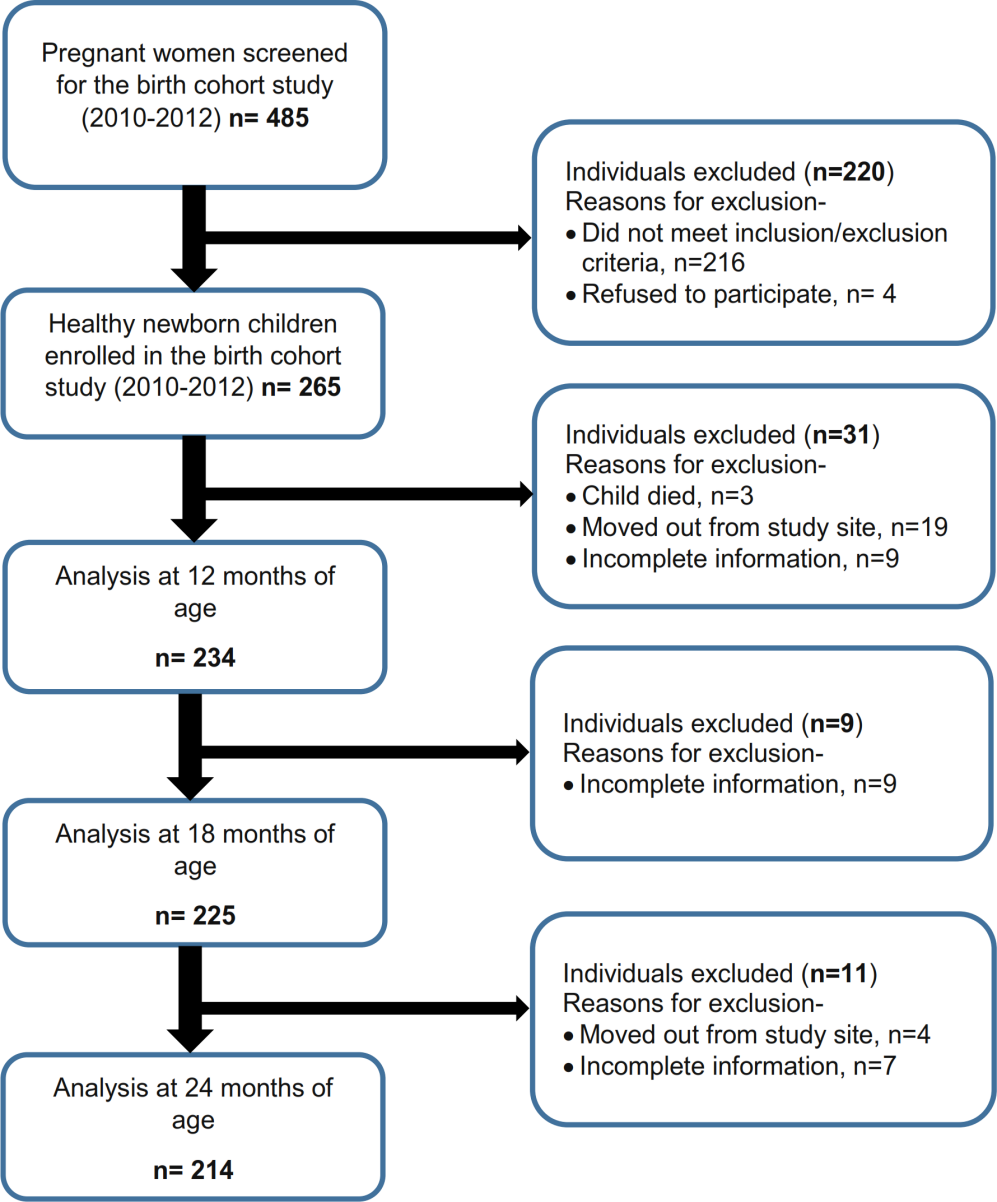
Flow diagram of the cohort study.

Total 265 children (130 males and 135 females) were enrolled in this study (Table 3). However, we have complete information on 234, 225 and 214 children at 12, 18 and 24 months of age respectively (Fig 1). The prevalence of LBW was 28.7%, and female children had 2-fold greater odds (OR = 2.3, 95% CI 1.32–4.0) of being low birth weight compared to male children. Considering LAZ, 21 male and 27 female (total 18%) were stunted at birth. At birth, mean LAZ was -1.08, mean WAZ was -1.31 and mean WLZ was -0.96. The mean LAZ of the participants further worsened with age and was -1.65, -1.95 and -2.03 at 12, 18 and 24 months, respectively. By 24 months of age, almost half of all the children (47.9%) became stunted (Table 3). No statistically significant association was found between gender and stunting status at any particular age using bivariate analysis (Chi-Square test). The average age of the mothers was approximately 25 years and 33% of them had completed primary education. Additional socio-economic characteristics of the study population are presented in Table 4.

**Table 3:** Anthropometric measurements of the children at different ages.

	Mean LAZ^a^	Mean WAZ^b^	Mean WLZ^c^	Stunting^d^ (%)	LBW^e^ (%)
At birth (n = 265)	-1.08±1.02	-1.31±0.92	-0.96±1.06	18.1	28.7
At 12 months (n = 234)	-1.65±0.93	-1.20±1.03	-0.51±1.01	33.6	
At 18 months (n = 225)	-1.95±0.93	-1.50±1.01	-0.76±0.99	46.8	
At 24 months (n = 214)	-2.03±0.93	-1.61±0.98	-0.75±0.92	47.9	

n = Number of participants

^a^ LAZ- Length-for-age Z-score

^b^ WAZ- Weight-for-age Z-score

^c^ WLZ- Weight-for-length Z-score

^d^ Stunting defined as height or length-for-age Z score (LAZ) more than two standard deviations below the

median of WHO Child Growth Standards

^e^ LBW- Low birth weight defined as weight at birth of less than 2,500 g (up to and including 2,499 g)

**Table 4:** Background characteristics of the study population.

Characteristics^a^	n = 265
Age of mother, y	24.92 ± 4.98
**Education level of mother, %**	
No schooling	19
Primary incomplete (1–5 y)	47
Primary completed (6–10 y)	33
Secondary completed or higher	1
Duration of the family has lived in house, y	2.8 ± 1.39 y
No. of room(s) in the household	1.62 ± 0.96
**Monthly household income, %**	
≤5,000 BDT^b^ (US$62)	21
5,001–10,000 BDT (US$62–$123)	54
10,001–15,000 BDT (US$123–$185)	14
>15,000 BDT (>US $185)	11
**Main source of drinking water, %**	
Piped into dwelling	19
Piped to yard or plot	81
Treat water to make it safe, %	64.3
**Improved toilet**^**c**^**, %**	
Yes	84.3
No	15.7

Continuous data are presented as the mean±SD and categorical data as proportion (%)

^a^ Characteristics during enrollment

^b^ BDT- Bangladeshi taka

^c^ Improved toilet- Toilets with a flush system to septic tank/ sewer and pit latrine with slab have been categorized as improved toilet

Average vitamin and mineral intakes between 9–12, 15–18 and 21–24 months and overall at 12–24 months of age have been summarized in Table 5. The recommended dietary allowance of each of the micronutrient and the calculated NAR values have also been provided in the same table. The daily recommended intake of Vitamin A is 300 microgram (μg) per day. However, we found that the mean daily intake in our cohort between 9–12, 15–18 and 21–24 months of age was 43.61 μg, 59.26 μg, and 80.20 μg respectively. Similarly, compared to the recommended intake of folate of 150 μg/d, the mean intake between 9–12 months of age was 21.02 μg/day, which increased to 36.21 μg/day between 15–18 months and 51.71 μg/day between 21–24 months of age. The average intake of vitamin E was 0.30 mg, 0.58 mg and 0.96 mg per day between 9–12, 15–18 and 21–24 months of age respectively, compared to the recommended value of 6 mg/day. In case of minerals, the average calcium intake between 9–12 months of age was 89.12 mg/day compared to the recommended 700 mg/day which increased to 104.42 mg/day between 15–18 months and 136.10 mg/day between 21–24 months of age. The RDA value of zinc is 3 mg/day for children 12–36 months of age. In contrast, the average intake of zinc in our study participants was found to be 0.78 mg, 1.14 mg and 1.68 mg per day between 9–12, 15–18 and 21–24 months of age, respectively.

**Table 5:** Average micronutrient intakes* from complementary food and the individual nutrient adequacy ratios at different ages.

	RDA^1^	9–12 mo (n = 234)	NAR^2^ 12	15–18 mo (n = 225)	NAR 18	21–24 mo (n = 214)	NAR 24	12–24 mo (n = 232)	NAR 12–24
**Vitamin A (μg/d)**	**300**	43.61±50.10	0.15	59.26±47.82	0.20	80.20±56.98	0.27	63.22±40.17	0.21
**Thiamine (mg/d)**	**0.5**	0.11±0.07	0.22	0.18±0.08	0.35	0.27±0.10	0.53	0.20±0.07	0.40
**Riboflavin (mg/d)**	**0.5**	0.17±0.18	0.35	0.24±0.17	0.47	0.32±0.22	0.63	0.25±0.16	0.50
**Niacin (mg/d)**	**6**	1.54±0.75	0.26	2.47±0.95	0.41	3.84±1.30	0.64	2.84±0.95	0.47
**Vitamin B6 (mg/d)**	**0.5**	0.17±0.10	0.34	0.25±0.12	0.51	0.35±0.14	0.70	0.27±0.10	0.55
**Folate (μg/d)**	**150**	21.02±13.00	0.14	36.21±19.97	0.24	51.71±21.83	0.34	39.36±16.07	0.26
**Vitamin B12 (μg/d)**	**0.9**	0.38±0.50	0.43	0.45±0.41	0.50	0.59±0.55	0.65	0.49±0.40	0.54
**Vitamin C (mg/d)**	**15**	7.06±9.07	0.47	12.65±16.69	0.84	18.07±20.32	1.20	13.01±10.24	0.87
**Vitamin E (mg/d)**	**6**	0.30±0.19	0.05	0.58±0.34	0.10	0.96±0.46	0.16	0.67±0.30	0.11
**Calcium (mg/d)**	**700**	89.12±131.05	0.13	104.42±103.76	0.15	136.10±140.30	0.19	110.64±107.10	0.16
**Iron (mg/d)**	**7**	0.92±0.68	0.13	1.38±0.70	0.20	2.16±1.25	0.30	1.59±0.77	0.23
**Zinc (mg/d)**	**3**	0.78±0.58	0.26	1.14±0.57	0.38	1.68±0.76	0.56	1.27±0.57	0.42
**Copper (μg/d)**	**340**	121.77±66.66	0.36	199.01±85.31	0.59	303.25±111.65	0.89	224.27±78.73	0.66
**MAR**^**3**^	**1**		0.24		0.35		0.48		0.39

*Intakes are presented as the mean ± SD

^1^ RDA- Recommended dietary allowance

^2^ NAR- Nutrient adequacy ratio calculated as the mean daily intake/RDA

^3^ MAR- Mean adequacy ratio calculated as ∑NARs/13 (when NAR>1, it was truncated to 1 before the calculation of the MAR)

For most of the vitamins and minerals, the corresponding mean NAR value was well below the optimum intake, which is 1. Among all the micronutrients, only the mean NAR of vitamin C reached that value between 21–24 months of age (Table 5). Vitamin E had the lowest mean NAR value at all the time points, which was 0.05, 0.10 and 0.16 between 9–12, 15–18 and 21–24 months of age respectively, and overall 0.11 between 12–24 months of age. The MAR, which is the mean value of all 13 NARs (truncated to 1), was 0.24 between 9–12 months of age. The MAR increased to 0.35 between 15–18 months and 0.48 between 21–24 months of age. Diet covering all the recommended nutrient intake should have an MAR value of 1, which is optimum. However, in our study, we found that MARs between 9–12, 15–18 and 21–24 months of age differed significantly from that ideal value (one-sample t-test, *p* = 0.000).

We used generalized estimating equation regression to explore any association between micronutrient adequacy and stunting. The results of unadjusted and adjusted GEE models are given in Table 6. We found that the MAR (%) was not a predictive factor of stunting among our study participants. In the final GEE model, age, gender, and history of LBW were the significant predictors of stunting between 12–24 months of age. Children with a history of LBW had 3-fold greater odds (AOR = 3.03, 95% CI: 1.69, 5.44) of being stunted compared to children with a normal birth weight. Male children had 2-fold greater odds (AOR = 1.98, 95% CI: 1.17, 3.33) of being stunted compared to female. With increasing age, the odds of being stunted almost increased by 2 folds at both 18 and 24 months of age (AOR = 1.97, 95% CI: 1.49, 2.59 and AOR = 2.12, 95% CI: 1.45, 3.11, respectively) compared to 12 months of age.

**Table 6:** Factors associated with stunting between 12–24 months of age and results from the generalized estimating equation (GEE) models^a^.

Variables^b^	Unadjusted OR^c^ (95% CI^d^)	*p-value*	Adjusted OR (95% CI)	*p-value*
**Age**				
24 month	**1.76 (1.38, 2.25)**	0.000	**2.12 (1.45, 3.11)**	0.000
18 month	**1.71 (1.36, 2.17)**	0.000	**1.97 (1.49, 2.59)**	0.000
12 month	Ref			
**Sex**				
Male	1.32 (0.83, 2.10)	0.244	**1.98 (1.17, 3.33)**	.011
Female	Ref			
**Currently breastfed**				
Yes	**0.52 (0.33, 0.83)**	0.006	0.62 (0.36, 1.08)	0.091
No	Ref			
**Mean adequacy ratio (%)**	**1.01 (1.00, 1.02)**	0.015	0.99 (0.98, 1.01)	0.537
**Had diarrhea in past 15 days**				
Yes	0.99 (0.80, 1.23)	0.928	1.14 (0.88, 1.47)	0.335
No	Ref			
**Maternal age**	1.00 (0.96, 1.05)	0.861	0.96 (0.89, 1.03)	0.221
**Birth order**	1.12 (0.91, 1.39)	0.271	1.28 (0.95, 1.72)	0.102
**Low birth weight (LBW)**				
Yes	**2.82 (1.67, 4.79)**	0.000	**3.03 (1.69, 5.44)**	.000
No	Ref			
**Presence of improved toilet**				
Yes	0.99 (0.54, 1.82)	0.977	0.80 (0.38, 1.68)	0.554
No	Ref			
**Water source**				
Piped to dwelling	**2.18 (1.15, 4.12)**	0.017	1.66 (0.82, 3.35)	0.159
Piped to plot	Ref			
**Mother’s education level**	0.93 (0.86, 1.00)	0.058	0.96 (0.88, 1.05)	0.361
**Household asset index**				
Poor	**1.90 (1.14, 3.18)**	0.014	1.61 (0.90, 2.87)	0.111
Intermediate	0.65 (0.32, 1.29)	0.213	0.55 (0.27, 1.11)	0.097
Wealthy	Ref			

^a^ Included repeated observations from 9–12 (n = 234), 15–18 (n = 225) and 21–24 (n = 214) months of age

^b^ Predictor variables included in the GEE model

^c^ OR-Odds ratio, significant findings are highlighted in bold

^d^ CI- Confidence interval

## Discussion

Our findings suggest that the prevalence of stunting is high among children less than two years old living in an urban slum area in Dhaka, Bangladesh. It further suggests that the dietary micronutrient adequacy is poor among the study population considering most of the micronutrients included in the analysis. However, we did not find any evidence suggesting a plausible causal association between poor micronutrient adequacy and stunting among the studied children between 12–24 months of age. Having a history of LBW was attributed as the prominent risk factor for the development of stunting among these children after adjusting for other predictors in a slum area of Bangladesh.

According to Bangladesh Demographic and Health Survey 2014, an alarming proportion of 36% children under-5 years of age are stunted with the prevalence being as high as 46% between 18–23 months of age [16]. As per the report of Bangladesh National Micronutrient Survey conducted in 2011–12, 32.1% of preschool children were stunted. However, the prevalence was much higher (51.1%) among the slum-dwelling children [44] suggesting that the burden of stunting is greater among slum dwellers which coincides with our study findings. Stunting is recognized as an accurate marker of societal inequities. Different studies from the countries with a high burden of childhood stunting report low socio-economic status, poor hygiene and sanitation, lack of maternal education and lack of access to improved water sources as substantial predictors of stunting [45–47]. Almost all these factors are universally present in the slum-dwelling population of Bangladesh which may explain such high prevalence of stunting.

Among our study participants, the average dietary consumption of most of the vitamins, such as vitamins A, E and the B complex and minerals, such as calcium, iron, and zinc is insufficient compared to international recommended value. Arsenault et al. [20] reported similar findings from a study in Bangladeshi rural population, where almost all the young children (age 24–48 months) and the women had inadequate intakes of vitamin A, riboflavin, folate, vitamin B12, and calcium. However, the average daily micronutrient intakes reported in that study were greater than what we found in our study in terms of majority of the vitamins and minerals at 24 months of age (vitamin A 117 vs. 80.2 μg/d, folate 65 vs. 51.7 μg/d, vitamin B12 0.8 vs. 0.6 μg/d, iron 3.6 vs. 2.2 mg/d, zinc 2.5 vs1.7 mg/d, calcium 145 vs. 136 mg/d). The difference in intakes may be attributable to the variance in the individual predisposition of the populations of interest. Studies conducted in Bangladesh [48] and similar LMICs [49,50], provide considerable evidence denoting impaired nutritional status of slum-dwelling children compared to rural community. The variation could also be due to the difference in research methodologies. The previous study measured dietary intake using a 12-hour direct observation followed by a 12-hour dietary recall multiple-pass method on 2 non-consecutive days. In contrast, the data in our study is based on reports from four 24-hour dietary recalls, which should be more representative of a typical intake.

We report the MAR as an overall measure of the nutritional quality of a child’s diet. The maximum MAR is only 48% at the age of 24 months among our study participants. Previously Arsenault et al. described a similar approach [20] to report nutritional quality of the diet among their study participants. They reported a probabilistic approach [51], where the probability of adequacy (PA) was calculated for each nutrient ranging from 0 to 1. They further calculated overall mean PA (MPA) by averaging the PA across all the 11 nutrients, similar to the calculation of the MAR. In that study, the overall prevalence of micronutrient intake adequacy was as low as 43% among children, similar to our study result. They concluded that low food energy intake and poor dietary diversity were the key factors behind such low micronutrient adequacy. Similarly, another study carried out among breastfed infants (6–12 months) in rural Bangladesh, reported that the complementary diet was deficient in comparison to the recommended intakes for multiple vitamins and minerals due to low micronutrient density [19]. Ensuring nutrient adequacy during the complementary feeding period is a major global health priority as inadequate intake, coupled with frequent infection causes permanent damage to already vulnerable children. Unfortunately, meeting the nutritional needs of 6-24-month-old children is challenging [8] due to dietary quality rather than quantity [52]. This is specifically applicable for LMICs where complementary foods are often nutritionally inadequate and infants are typically fed diluted plant-based family foods, hence low in nutrients [8,53]. Similar dietary practice might explain the overwhelming poor micronutrient quality of the diet among the children in our study.

However, we did not find poor micronutrient adequacy as a significant predictor of stunting among the studied children after adjusting for other important explanatory variables. This is likely due to an overall poor micronutrient adequacy in the general population that affects both stunted and non-stunted children equally. In a Bangladeshi study that was carried out in a similar slum context, complementary food intake was not found to exhibit a significant impact on growth [54]. A similar result was reported by Theron et al. from their NutriGro study [55], in which inadequate dietary intake was not found to be a causal factor of stunting amongst young children living in an urban informal settlement in South Africa. Another Zambian study [56] compared the median nutrient intake from weaning food for both stunted and non-stunted infants and observed no conspicuous differences between the study groups. All of these studies reported an overall poor quality of the complementary diet with low nutrient content similar to the scenario in our study. This suggests that both stunted and non-stunted children involved in the study consume a nutrient deficient diet to an identical extent.

Our findings also illustrate a greater prevalence of LBW in our study compared to that from recent national data (29% vs. 22%) [57]. The difference in the prevalence might be due to the difference in the context of the slum population. This was demonstrated by another study [54] which also reported a higher prevalence (46.4%) of LBW in Bangladeshi slums. Moreover, we report LBW as a significant determinant of stunting in our study population. Several other studies conducted in a similar context reported comparable results where LBW was found to be a substantial risk factor for childhood mortality and morbidity [58–61]. Consequently, it has been documented that approximately 30% children with LBW remain of a short stature [62–64]. Arifeen and colleagues reported that birth weight was the key determinant of subsequent growth status during infancy [59] based on their independent study conducted in a Bangladeshi slum area.

It is well established that, in the developing countries, LBW is often associated with intrauterine growth restriction (IUGR) and stunting [46]. IUGR is regarded as the failure of a fetus to achieve the actual genetic growth potential due to nutritional deprivation during gestation or due to other environmental factors [65]. Infants born with LBW continue to experience growth failure during early childhood, which might persist until adolescence. Evidence suggests that there is a strong intergenerational relationship between birth weight and subsequent gain of length as rationalized by both genetic and environmental influences across generations [66]. Environmental influences have also been suggested to have substantial impact in poverty-stricken settings, such as that in slum areas. According to the “Barker hypothesis”, [67] certain diseases have been suggested to originate through adaptations of the fetus when it is undernourished [68]. Such adaptations may cause permanent structural and/or functional changes to the body to facilitate survival over growth and thus, may explain the strong association between LBW and stunting in LMICs like Bangladesh.

Considering this context, our study result has judicious policy implications. Despite a marked improvement in other health-related indicators, Bangladesh is struggling to tackle the burden of chronic malnutrition. Once a baby is born underweight, the risk of becoming undernourished increases by many folds despite a suitable environment [61]. Our study results thus suggest that context-specific strategies should be formulated in order to curtail growth faltering and subsequent negative health outcomes.

## Strengths and limitations of the study

The key strength of our study findings is the study design and subsequent statistical analysis. The majority of the studies that aim to identify nutrient adequacy and its association with growth faltering have been conducted using a cross-sectional design at a single time point. However, in our study, due to the cohort design, we have measured and subsequently accumulated all the information multiple times prospectively, indicating the temporal sequence between the predictor and outcome variables. Such repeated measurement enabled us to estimate and report the typical dietary intake in the population. In our statistical analysis, we used the appropriate method to account for repeated measurement of multiple variables of interest, which has further strengthened our study findings.

However, one of the key limitations of our study is the sample size. The measurement of micronutrient intake is small, and hence identification of any extent of difference between the groups requires a large sample size, which was beyond the scope of this study, taking into consideration the study design and required resources. Therefore, this study may not have sufficient power to identify any true association between micronutrient adequacy and stunting.

Furthermore, the collection of breast milk was beyond our scope provided by the settings of the study. Breast milk composition is one of the most decisive factors when drawing any conclusion regarding nutrient adequacy, particularly for breastfed children. The nutrient value of several micronutrients (vitamin A, riboflavin, thiamine, and vitamins B_6_ and B_12_) depends on the mother’s nutritional status [19] whereas folic acid, calcium, iron, copper, and zinc are less likely to be affected by the nutritional condition of the mother [9]. However, a study [20] conducted among Bangladeshi women reported that micronutrient intake adequacy in women is even lower than that of the children due to inadequate intake and limited diversity, which can be explained by their dependence on the plant-based diet. Considering the low nutrient adequacy of the complementary diet in our study, breast milk quality might not be sufficient to meet the requirement of these children.

## Conclusion

The degree of micronutrient adequacy of the complementary food is deficient for most vitamins and minerals among children below two years of age in a slum-dwelling population of Bangladesh. However, such inadequacy does not account for the high prevalence of stunting that was observed in the study. Rather, history of low birth weight is the prominent determinant of stunting among these slum-dwelling children, after adjusting for other factors. Improving the nutritional quality of the complementary food is imperative for optimum growth. However, this may not be sufficient enough to mitigate the burden of stunting in impoverished slums. Further research should focus on identifying multiple strategies that could work synergistically to diminish the burden of stunting in such underprivileged and resource depleted settings.

## Supporting information

S1 File STROBE (Strengthening The Reporting of Observational Studies in Epidemiology) checklist. (PDF)
